# A Survey of Therapeutic Radiographers’ Knowledge, Practices, and Barriers in Delivering Health Behaviour Advice to Cancer Patients

**DOI:** 10.1007/s13187-020-01896-x

**Published:** 2020-10-15

**Authors:** Nickola D. Pallin, Rebecca J. Beeken, Kathy Pritchard Jones, Nick Woznitza, Abigail Fisher

**Affiliations:** 1 Department of Behavioural Science and Health, University College London, London, UK; 2 Department of Allied Health Sciences, School of Health and Social Care, London South Bank University, London, UK; 3 Leeds Institute of Health Sciences, University of Leeds, Leeds, UK; 4 University College London Partners Academic Health Science Network, London, UK; 5 Radiology Department, Homerton University Hospital, London, UK

**Keywords:** Health behaviours, Healthy lifestyle, Therapeutic radiography, Radiation therapy, Advice provision

## Abstract

Therapeutic radiographers (TRs) are well-placed to deliver advice to cancer patients; however, limited research exists on their practices in providing advice on healthy lifestyle behaviours. Through an online survey, this study aimed to explore TRs’ current practices, barriers, and facilitators around delivering healthy behaviour advice to cancer patients. An online survey was sent to 72 radiotherapy departments in the UK and 583 TRs responded to the survey. Findings showed that levels of enquiry and provision of advice on healthy behaviours were low, with less than 25% advising patients on physical activity, healthy eating, weight management, smoking cessation, and reducing alcohol intake as standard practice. Lack of knowledge, resources, and training were identified as barriers, in addition to perceived lack of patient interest and lack of time. TRs reported a strong desire to undergo training to enable them to deliver health behaviour advice to patients, with an identified preference for online training. Cancer patients look to healthcare professionals for advice on health behaviours, and TRs are well-placed to deliver this advice. The findings of this study provide insight into the areas that need addressing to enable TRs to support positive health behaviours among cancer patients.

**Electronic supplementary material:**

The online version of this article (10.1007/s13187-020-01896-x) contains supplementary material, which is available to authorized users.

## Introduction

Healthcare providers are expected to deliver advice on healthy eating, weight control, physical activity (PA), limiting alcohol consumption, and reducing smoking to all cancer patients to reduce their risk of secondary cancers and comorbidities [1]. However, whilst research shows that some oncology healthcare professionals (HCPs) offer guidance to oncology patients on healthy lifestyle changes, provision remains suboptimal [2–6]. In the UK, a qualitative study among ten oncology HCPs and sixteen prostate cancer patients found that HCPs do not routinely provide advice on diet and PA to men diagnosed with prostate cancer, with some patients unable to recall receiving any advice on diet or PA from their healthcare team [3]. Reported barriers among oncology HCPs in delivering advice on health behaviours include believing that giving advice is not part of their role, lack of support and time, lack of guidelines, lack of knowledge of the evidence base, and concerns around seeming to blame the patient [2–6].

At least 50% of those with a cancer diagnosis undergo radiotherapy as part of their treatment [7]. Therapeutic radiographers (TRs), also known as radiation therapists, are uniquely placed to deliver advice on healthy lifestyle behaviours to cancer patients. Patient education is a key part of radiotherapy practice, with TRs providing care to the same patient every day, often over a number of weeks [8]. Despite this, there has been less research among TRs and the delivery of health behaviour advice. Only one study has been undertaken in the UK that investigated the current provision of advice on health behaviours including smoking, alcohol, healthy eating, and exercise [5]. This study, through a survey (*n* = 102), found that levels of advice provided to patients on these topics were low [5]. No research has been undertaken to assess TRs’ barriers to providing health behaviour advice for cancer patients, and how to address these. Therefore, this study aimed to explore TRs’ current practices, knowledge, barriers, and facilitators around delivering healthy behaviour advice to cancer patients. Therapeutic radiographers’ needs and preferences in terms of training on this topic were also explored.

## Methods

### Participants and Recruitment

TRs and assistant practitioner TRs in the UK working within the cancer care setting were invited to take part in an online survey. Practising TRs in the UK have completed a recognised degree to meet the standards of proficiency for a band 5 agenda for change (AfC) radiographer and are registered with the Health and Care Professions Council (HCPC) [9]. An assistant practitioner is a non-registered practitioner but performs protocol-limited clinical tasks under the direction and supervision of a state-registered TR practitioner, and works within a band 4 AfC definition [10]. In the UK, the professional grade of TRs is defined by AfC, from band 5 to band 8 which reflect an individual’s professional skills, responsibilities, and job-related knowledge [10]. From August 2018 to April 2019, a link to an online survey was emailed to 72 radiotherapy departments in the UK and cascaded through the mailing lists within each department. Participants were made aware that by completing the survey, they were providing informed consent for the use of their data for this research. This study was approved by University College London’s ethics committee, reference 12945/001.

### Measures

The survey questions were based on a previous study carried out by Williams et al. [2]. The survey (Supplementary Material) was adapted for use among TRs, with additional questions added to identify the practices in delivering advice on alcohol consumption and sun safety. The questionnaire was piloted among a group of TRs working clinically (*n* = 2) and in the academic sector (*n* = 2). As a result of the feedback, one question designed to explore the delivery of advice on healthy eating, but which used the term ‘diet’, was amended to ‘healthy eating’. Respondents were asked to provide their professional grade, to allow for comparison with public statistics on the profile of TRs in the UK. The UK radiotherapy radiography workforce census only reports the workforce’s AfC band, also known as professional grade, and do not report any other demographics [11]. Therefore, to allow for comparison with public statistics, respondents were asked to provide their AfC band and no other demographics were collected.

### Analyses

The survey responses were analysed using descriptive statistics with the statistical package SPSS version 25. Missing data were recoded as ‘unknown’ for analyses to include as many respondents as possible. To assess whether awareness of lifestyle guidelines was related to the provision of advice on each health behaviour, a multinomial logistic regression model was carried out. The dependent variable was each level of advice provision (advice provided to no patients/advice provided to 1–25%/26–50%/51–75%/> 75% of patients). Respondents’ professional grade was added as covariates to each of these models. Data obtained from the open-ended questions were transferred into qualitative data analysis software (NVivo, version 12) and coded line by line. The open responses from each question were grouped together and analysed to identify any patterns or themes. A deductive approach to analysis was undertaken using the content analysis process [12]. Themes are presented in the results with supporting quotes and the participant’s AfC band.

## Results

### Response Rate

The exact response rate is unknown because the link to the survey was cascaded independently within each radiotherapy department. However, according to the 2019 census of the UK radiotherapy workforce [11], there are 3392 TRs in the UK. The survey was started by 583 TRs who answered at least one question, and therefore, the responses are representative of 17% of the radiotherapy workforce in the UK. Of these 583, 367 (63%) completed all of the survey questions. The results are reported in percentages of those who answered the corresponding question. In total, 662 individual open-response qualitative comments were analysed.

### Agenda for Change Band

One percent (*n* = 6) were band 4 assistant practitioner TRs, 23% (*n* = 128) were band 5 TRs, 31% (*n* = 174) were band 6, 30% (*n* = 168) were band 7, and 15% (*n* = 88) were band 8 TRs.

### Beliefs on Role Responsibilities

Eighty-nine percent (*n* = 399) thought providing healthy lifestyle advice was part of their role. Of those who said no (11%; *n* = 51), analysis of the open-ended questions showed that the main reasons were believing that advice provided within their role should only be related to the impact on radiotherapy delivery or radiotherapy–related side effects. For example, one respondent commented ‘Give advice on how it can impact treatment, i.e. smoking, but not on alternative to stop smoking etc. … just to stop 2 hours before and after RT’ (Band 6 TR). Other reasons were lack of knowledge, lack of time, and believing that the delivery of lifestyle advice is the responsibility of other members of the multidisciplinary team (MDT). One respondent commented ‘I feel it is not specific to our role to provide healthy lifestyle advice. There are more specially trained members of the MDT’ (Band 6 TR).

### Beliefs on Having the Skills and Knowledge to Deliver Health Behaviour Advice

Of all the health behaviours, sun safety was the topic that most TRs (80%; *n* = 323) felt they had the skills and knowledge to deliver advice on, followed by PA (59%; *n* = 238), smoking cessation (54%; *n* = 218), healthy eating (53%; *n* = 214), and alcohol intake (52%; *n* = 210). Providing advice on weight management was the topic respondents felt they had the least amount of skills and knowledge to deliver advice on (36%; *n* = 145). The main theme from the open-ended responses was TRs particularly felt unqualified and lacked knowledge of guidelines on the topic of healthy eating. One TR commented ‘I feel unqualified to give specific healthy eating advice with differing opinions on diets such as dairy free, red meat free etc.’ (Band 7 TR). Another respondent wrote ‘I am aware of what constitutes as healthy eating, but lack confidence in my knowledge of published guidelines’ (Band 7 TR).

### Levels of Enquiry

The proportion of patients seeking advice on health behaviours reported by the respondents is shown in Table 1. Patients seeking advice was highest for sun safety, followed by alcohol intake and healthy eating. Patients seek advice about smoking, weight management, and PA less frequently. Table 1 shows the proportion of respondents who reported enquiring about each health behaviour and providing advice. Less than 20% of TRs reported advising patients on healthy eating, weight management, smoking cessation, and reducing alcohol intake to more than 50% of their patients. Twenty-four percent reported advising patients on PA to more than 50% of their patients, with TRs enquiring and advising on sun safety more frequently than other health behaviours.

**Table 1:** Proportion of patients seeking health behaviour advice and TRs enquiring and advising on health behaviours

	None	1–50%	> 50%
The percentage of TRs reporting the percentage of patients seeking advice (*N* = 435)			
Physical activity	25%	67%	8%
Healthy eating	26%	62%	12%
Weight management	46%	47%	7%
Smoking cessation	44%	53%	3%
Alcohol intake	23%	62%	15%
Sun safety	25%	56%	19%
The percentage of TRs who report enquiring about health behaviours (*N* = 420)			
Physical activity	28%	48%	24%
Healthy eating	34%	47%	19%
Weight management	47%	39%	14%
Smoking cessation	27%	48%	25%
Alcohol intake	24%	53%	23%
Sun safety	17%	44%	39%
The percentage of TRs who report advising patients on health behaviours (*n* = 408)			
Physical activity	22%	54%	24%
Healthy eating	31%	52%	17%
Weight management	35%	47%	18%
Smoking cessation	29%	55%	16%
Alcohol intake	28%	54%	18%
Sun safety	11%	46%	43%

### Familiarity with Guidelines

Seventy six percent (*n* = 344) were aware of some guidelines for cancer patients on health behaviours. Awareness was highest for smoking cessation guidelines (69%; *n* = 312) and lowest for weight management guidelines (31%; *n* = 140). Sixty percent (*n* = 271) were aware of guidelines for sun safety (60%; *n* = 271), 52% (*n* = 235) were aware of guidelines for alcohol intake, 48% (*n* = 217) were aware of PA guidelines, and 44% (*n* = 199) were aware of healthy eating guidelines. Seventy percent (*n* = 315) of respondents were unable to recall the source of the guidelines. Of those who could recall a guideline, Macmillan Cancer Support was the most commonly mentioned resource (*n* = 55) followed by in house departmental guidelines (*n* = 23).

As shown in Table 2, awareness of guidelines for each health behaviour was associated with increased likelihood of TRs enquiring about patients’ lifestyle behaviours and providing advice for all health behaviours. The baseline was the provision of no advice on health behaviours. Awareness of guidelines was associated with increased likelihood of providing advice on PA to more than 75% of patients [odds ratio (OR) = 5.61; 95% confidence interval (CI) 2.57–12.3, *P* < 0.001], healthy eating [OR = 4.11 (95% CI 1.51–11.23), *P* < 0.01], weight [OR = 3.18 (95% CI 1.43–7.04), *P* < 0.01], smoking [OR = 3.13 (95% CI 1.26–7.79), *P* < 0.05], alcohol [OR = 2.61 (95% CI 1.19–5.75), *P* < 0.05], and sun [OR = 2.85 (95% CI 1.39–5.85), *P* < 0.001] (Table 2).

**Table 2:** Associations between awareness of health behaviour guidelines and level of enquiry and advice provision on each health behaviour

	Physical activity OR (95% CI)	Healthy eating OR (95% CI)	Weight management OR (95% CI)	Smoking cessation OR (95% CI)	Alcohol intake OR (95% CI)	Sun safety OR (95% CI)
Level of enquire about lifestyle (*N* = 420) Ref = none						
1–25%	1.11 (0.66–1.86)	1.00 (0.59–1.68)	2.09 (1.25–3.49)*	2.08 (1.26–3.46)*	1.3 (0.78–2.16)	2.36 (1.27–4.39)**
26–50%	2.28 (1.24–4.17)**	2.079 (1.18–3.66)	4.50 (2.28–8.92)***	1.97 (0.95–4.07)	2.14 (1.16–3.97)*	2.73 (1.44–5.16)**
51–75%	2.70 (1.34–5.46)**	4.03 (1.97–8.23)***	2.68 (1.21–5.98)*	2.05 (0.93–4.52)	2.09 (1.04–4.21)*	1.98 (1.01–3.87)*
> 75%	4.01 (1.99–8.07)***	5.44 (2.35–12.56)***	4.25 (1.81–9.97)***	5.35 (2.42–1.84)***	2.15(1.07–4.32)*	3.58 (1.87–6.87)***
Level of advice provision (*N* = 408) Ref = none						
1–25%	2.29 (1.29–4.07)*	1.94 (1.17–3.21)**	1.36 (0.75–2.44)	2.36 (1.44–3.88)***	1.53 (0.94–2.49)	2.67 (1.26–5.34)**
26–50%	5.52 (2.82–10.89)***	3.14 (1.68–5.87)***	3.71 (1.99–6.94)***	2.41 (1.19–4.92)*	2.87 (1.49–5.54)*	2.35 (1.12–4.94)*
51–75%	3.50 (1.78–7.23)***	3.26 (1.62–6.55)***	4.01 (1.98–8.48)***	5.86 (1.93–17.88)**	7.40 (3.01–8.22)***	2.38 (1.17–5.16)*
> 75%	5.61 (2.57–12.3)***	4.11 (1.51–11.23)**	3.18 (1.43–7.04)**	3.13 (1.26–7.79)*	2.61 (1.19–5.75)*	2.85 (1.39–5.85)**

A multinomial logistic regression model with awareness of lifestyle guidelines (Y/N) and level of enquiry and advice provision (none/1–25% of patients/26–50% of patients/51–75% of patients/> 75% of patients) as the dependent variable

Ref: Reference category no advice = provide advice to no patients

*OR* odds ratio, *CI* confidence intervals

*P < 0.05; **P < 0.01; ***P < 0.001

### Barriers to Providing Advice

Table 3 shows the reported barriers in providing health behaviour advice. Patients being too frail or ill were the most commonly reported barrier to delivering advice on PA (45%; *n* = 170) followed by perceived lack of patient interest (42%; *n* = 157), and not knowing the guidelines (41%; *n* = 156). Not knowing the guidelines was the most common barrier to delivering advice on healthy eating (44%; *n* = 166) and weight management (41%; *n* = 155). For smoking and alcohol intake, perceived lack of patient interest was the most frequently reported barrier in delivering advice on these topics, 45% and 41%, respectively. Lack of time as a barrier was commonly reported for all health behaviours (Table 3).

**Table 3:** Barriers among TRs in providing health behaviour advice (*N* = 378)

	Physical activity (%)	Healthy eating (%)	Weight management (%)	Smoking (%)	Alcohol (%)	Sun safety (%)
Not knowing the guidelines	41	44	41	25	31	22
Not knowing what to say	17	18	28	21	21	7
Lack of time	39	36	34	33	33	30
Do not think part of role	11	12	16	7	8	4
Don’t know where to refer patients to	29	17	23	19	25	19
Lack of patient interest	42	36	30	45	41	28
Seeming to blame patient	10	14	21	26	22	5
Not convinced it affects cancer outcomes	2	2	2	1	2	1
Patient being too frail or ill	45	24	25	25	21	19

Qualitative comments gave further insight into TRs’ barriers in delivering advice, most commonly lack of training and knowledge. ‘We are given very little training/guidance on how to approach and advise on these issues but I believe we should’ (Band 7 TR). One TR wrote ‘Topics aren’t covered at University level so the background knowledge and confidence to discuss isn’t there’ (Band 5 TR). Risk of patients’ changing body shape affecting the accuracy of radiotherapy treatment was also a reported barrier. ‘Maintaining healthy weight (losing weight or gaining weight) is not appropriate during treatment as weight should be maintained post CT scan in order to deliver accurate treatment, therefore I feel it is an issue to tackle afterwards’ (Band 5 TR). Additionally, one theme that emerged from the comments was the belief that advice on healthy eating may exacerbate treatment–related side effects ‘Healthy eating advice is not always appropriate for patients receiving treatment in certain areas e.g. for pelvis treatment where high fibre intake may exacerbate symptoms’ (Band 6 TR).

### Facilitators to Providing Advice

Of those who answered the question (*n* = 375), online training (73%; *n* = 273) was the most commonly requested support for facilitating the delivery of lifestyle advice. This was followed by the provision of referral pathways for lifestyle support (69%; *n* = 259) and education resources for patients within the department (64%; *n* = 239). Sixty-one percent (*n* = 229) reported that in house training (61%; *n* = 229) would be helpful, as well as role expansion (50%; *n* = 189) and mandatory continuous professional development (CPD) training (39%; *n* = 147). The qualitative comments from the open-ended responses further highlighted the role of training both in the pre-registration and post-registration setting in facilitating TRs’ to deliver health behaviour advice. ‘Inclusion in degree as part of advice giving during practical’ (Band 6 TR). Another TR wrote ‘Would be nice to have staff take a mandatory module to be able to personally provide the information with confidence’ (Band 7 TR). Preferred topics delivered within a training course were the current evidence for specific health behaviours and cancer outcomes (91%; *n* = 339), followed by information of available support and patient education resources (88%; *n* = 327), video examples of how to deliver advice (51%; *n* = 188), and role play of having a conversation with a patient (20%; *n* = 76). Open-ended responses further showed a preference for online training, mainly because of the difficulties associated with permitting all staff to attend face to face training. ‘Face to face would take a long time for all radiotherapy staff to be trained unless it was done at a lunchtime or out of hours with overtime to be claimed back. Part time staff may miss out if the training is not on their day to work’ (Band 6 TR). Another respondent wrote ‘Online training is good too, only if it is made mandatory’ (Band 7 TR).

## Discussion

The findings show that whilst the majority (89%) of TRs believe providing health behaviour advice to cancer patients is part of their role, this is not matched by provision of advice. These results are lower than reported in previous studies among oncology HCPs [2, 13]. One explanation may be that the participants within these studies were primarily oncologists and nurses. It is well-documented that doctors and nurses are expected to deliver nutrition and lifestyle advice [14, 15]. It is only in recent years that TRs have been recognised as a key healthcare member in delivering health behaviour advice [16] and this may explain why self-reported delivery of healthy lifestyle advice is higher among oncology HCPs in previous studies. Data collected from oncology HCPs have most commonly identified lack of knowledge, confidence, and skills as barriers to the delivery of health behaviour advice [2–4], in addition to lack of time, perception that patients lack interest, patient being too frail or ill, and believing they are not the right persons to provide advice [2–4]. This study confirmed these barriers among TRs and illustrated from the qualitative comments some TRs felt that lifestyle advice provided within their role should only be in relation to the management of radiotherapy treatment–related side effects.

Our qualitative data suggest that the low level of advice provision on weight management may be in part due to TRs not wanting patients to change their body shape as this can affect the accuracy of radiotherapy treatment. In addition, as reported in other studies [13], some HCPs may be hesitant to discuss sensitive topics such as weight management to avoid the risk of offending patients. Therefore, TRs may need support and guidance with initiating and managing potentially difficult conversations around lifestyle behaviour changes. In addition to identifying an appropriate time to initiate these conversations, there needs to be a balance between immediate treatment requirements and long-term survivorship needs.

Although it is well-known that continued smoking after a cancer diagnosis is associated with increased risk of cancer recurrence and higher mortality rates [17], advice on smoking cessation was low, with only 25% enquiring and 16% advising on smoking cessation to more than 50% of their patients. Perceived lack of patient interest was the highest reported barrier in delivering advice on smoking cessation. Likewise, in a UK study, this was reported as a barrier among TRs in providing smoking cessation support [6]. Considering that cancer patients show a desire and motivation to quit but often do not ask for help [18], TRs should be skilled in initiating a conversation around smoking cessation and referring patients for further smoking cessation support. This is particularly important because patients who attend smoke-free services are four times more likely to quit [19]. With perceived lack of patient interest a reported barrier, TRs need to be supported to use their role to initiate discussions regarding this behaviour as per recommendations [1].

Our qualitative data suggested TRs also felt they were particularly unqualified and lacked the skills in delivering healthy eating advice. Evidence is increasingly showing the relationship between dietary habits and cancer development and the role of a healthy diet in improving cancer survival [20]. Advice regarding nutrition benefits for both the general public and cancer survivors is often inconsistent and at times contradictory [21], and patients therefore need directing to information that is reliable and underpinned by high-quality evidence. This is particularly important given that 74% of TRs in our study estimated that their patients ask for information on healthy eating.

Less than 50% of respondents reported awareness of lifestyle guidelines for PA, healthy eating, and weight management for cancer survivors. Of those who could recall a guideline for lifestyle advice, Macmillan Cancer Support was the most commonly mentioned resource (*n* = 55). In a recent qualitative study, lack of knowledge of guidelines among oncology HCPS was also highlighted, with no HCP being able to name specific lifestyle guidelines for cancer survivors [4]. In the current study, TRs who were aware of guidelines for each health behaviour were also more likely to enquire and provide advice on each health behaviour. Similarly, in another study, awareness of guidelines was associated with increased likelihood of providing lifestyle advice [2]. This illustrates the need for wider dissemination of evidence-based guidelines on health behaviours and cancer survivorship in the radiotherapy department. This need is further recognised whereby 64% of TRs reported that the provision of education resources for patients within the department would support the provision of health behaviour advice within their role.

The reported barriers within this study around lack of knowledge and skills highlight the need for training and education. This is not surprising considering that education on providing health behaviour advice has not been an important component of the training of HCPs [22]. With the recent call for a radical upgrade in prevention in healthcare, it is expected that allied health professionals have the appropriate education and training to deliver advice to motivate people to make health behaviour changes [23]. Encouragingly, TRs reported a strong desire to undergo training to enable them in delivering health behaviour advice and a preference for online training was identified. From the qualitative comments, TRs recognised the benefit of online learning in allowing for mandatory continuous professional development. The provision of web-based health education for oncology HCPs has shown to be positive, with self-reported improvements in knowledge and practices on delivering nutrition and health advice following completion of an online training resource [24]. However, limited web-based training exists for oncology HCPs on the delivery of health behaviour advice to cancer patients, with no training available specifically for TRs. This study highlights the need for post-graduate training on health behaviour advice. However, the value of including training within the undergraduate education for TRs was highlighted, which has been identified as a requirement within the allied health professions pre-registration education recommendations in the UK [25]. Additionally, this study provides insight into TRs’ preferences on course content, with a particular desire for dissemination of the current evidence for specific health behaviours and cancer survivorship, followed by information of available support and patient education resources.

### Strengths and Limitations

This is the largest study to explore the practices, barriers, and facilitators among TRs in delivering health behaviour advice to cancer patients. The results provide insight into the practices among TRs across all AfC professional grades. The respondents from each AfC grade are also representative of the UK radiotherapy radiography workforce [11]. In the UK, 2%, 26%, 35%, 26%, and 10% are AfC bands 4, 5, 6, 7, and 8, respectively, similar to the distribution of respondents in this study. The large number of qualitative comments also provided a range of views, adding further insight into TRs’ beliefs on the delivery of health behaviour advice within their role. However, there are a number of limitations. Although all radiotherapy departments were invited to participate, it is unknown if the responses are representative of all radiotherapy departments in the UK. Additionally, the respondents may also have been more motivated to respond due to already having an interest in healthy lifestyle behaviours, which may mean TRs are less likely to provide health behaviour advice than those who completed the survey.

## Conclusion

The findings of this study show that the provision of health behaviour advice among TRs is suboptimal, despite recognition that this is part of their role. There is a clear need for training and improved education among TRs in order to enhance their delivery of health behaviour advice. It is also vital to support TRs in delivering advice and subsequently increasing the number of cancer patients receiving advice on improving health behaviours.

## Electronic supplementary material

ESM 1 (PDF 273 kb)

## Data Availability

Data can be obtained by the corresponding author on reasonable request.
