# Supplementary material for: A Survey of Therapeutic Radiographers’ Knowledge, Practices, and Barriers in Delivering Health Behaviour Advice to Cancer Patients
Source: J Cancer Educ. 2020 Oct 15;37(4):890–7. doi: 10.1007/s13187-020-01896-x (PMC9399055; doi:10.1007/s13187-020-01896-x)
Supplement: Supplementary file 1 — (PDF 273 kb) [file 13187_2020_1896_MOESM1_ESM.pdf]

Health Promotion among Radiographers

Identifying how Therapeutic Radiographers can be supported to deliver healthy lifestyle advice

We would like to invite you to complete this brief survey about your views on the current practices among therapeutic radiographers in providing lifestyle advice and how you can be supported to promote healthy lifestyle behaviours among cancer patients. Your responses will help highlight areas for improvement and also help inform the development of a training intervention for therapeutic radiographers on delivering lifestyle advice to cancer patients.

The survey contains 20 questions and should take no more than 10 minutes to complete.

To be eligible to take part you must meet the following criteria:

Be a Therapeutic Radiographer or Radiotherapy Assistant Practitioner.

You are free to withdraw at any time without giving a reason.

All data will be collected and stored in accordance with the Data Protection Act 2018 and will not be shared or used for any other purpose.

If you would like more information please contact the researchers using the contact details provided. We can provide you with a summary of the findings on request.

Thank you for your interest in taking part in this research. If you have any questions please contact the researchers on XXXX

We need to request formal consent so please could you read the following statements and check the box below if you agree to take part.

1) I have read the study information and understand what it involves.
2) I understand that if I decide at any time that I no longer wish to take part I can exit from the survey.
3) I consent to the processing of my personal information for the purpose of this research study.
4) I agree that the research project named above has been explained to me to my satisfaction and I agree to take part in this study.

* 1. I have read the above statements and I agree to complete this survey

☐ Yes

Health Promotion among Radiographers

* 2. Please state your agenda for change banding.

- 4
- 5
- 6
- 7
- 8 or above

Health Promotion among Radiographers

* 3. Do you think providing healthy lifestyle advice to cancer patients is part of your role?

- Yes
- No

If you selected NO please explain why

* 4. Are you aware of any guidelines specifically for cancer patients for any of the following lifestyle topics? Please select all that apply.

- Physical Activity
- Healthy Eating
- Weight Management
- Smoking
- Alcohol Consumption
- Sun Safety
- I am not familiar with any guidelines on these topics
- Other (please specify)

* 5. Do you know the name of any lifestyle guidelines for cancer? Pleased list all of the guidelines and information that you are aware of

* 6. Can you estimate how many of your patients Ask You for information about the following lifestyle topics?

	none	1-25%	26-50%	51-75%	>75%
Physical Activity					
Healthy Eating					
Weight Management					
Smoking					
Drinking Alcohol					
Sun Safety					

Other (please specify)

* 7. Thinking of all your patients who have completed radiotherapy treatment, how many of your patients do you ask about the following lifestyle topics? By this we mean ask patients directly about lifestyle (e.g. 'Do you smoke?' or 'Are you regularly active?')

	None of the time	1-25%	26-50%	51-75%	>75%
Physical Activity	○	○	○	○	○
Healthy Eating	○	○	○	○	○
Weight Management	○	○	○	○	○
Smoking	○	○	○	○	○
Drinking Alcohol	○	○	○	○	○
Sun Safety	○	○	○	○	○

Other (please specify)

* 8. Again thinking of all your patients who have completed radiotherapy treatment, how many of your patients did you advise on any of the following lifestyle topics? By advise we mean give direct advice or referral.

	None	1-25%	26-50%	51-75%	>75%
Physical Activity	◯	◯	◯	◯	◯
What constitutes a healthy diet	◯	◯	◯	◯	◯
Importance of maintaining a healthy weight	◯	◯	◯	◯	◯
Smoking cessation	◯	◯	◯	◯	◯
Reducing alcohol intake	◯	◯	◯	◯	◯
Sun Safety	◯	◯	◯	◯	◯

* 9. Do you feel you have sufficient skills and knowledge to give patients up to date lifestyle and health promotion advice on the following topics?

	Yes	No
Physical Activity		
Healthy eating advice		
Weight management advice		
Smoking cessation		
Limiting Alcohol intake		
Sun Safety		

Please comment

* 10. What circumstances prevent you from providing advice about any of these topics? Please select all that apply

	Not knowing the current guidelines	Not knowing what to say	Lack of time	Do not think it is part of my role	Don't know where to refer patients	Lack of patient to interest	Seeming to blame the patient	I'm not convinced it affects cancer outcomes	Patient being too frail or ill
Physical activity	☐	☐	☐	☐	☐	☐	☐	☐	☐
Healthy eating	☐	☐	☐	☐	☐	☐	☐	☐	☐
Weight management	☐	☐	☐	☐	☐	☐	☐	☐	☐
Smoking	☐	☐	☐	☐	☐	☐	☐	☐	☐
Drinking alcohol	☐	☐	☐	☐	☐	☐	☐	☐	☐
Sun Safety	☐	☐	☐	☐	☐	☐	☐	☐	☐

* 11. Can you think of any other barriers within your role and work environment stopping you from giving lifestyle and health promotion advice to patients?

Health Promotion among Radiographers

* 12. What support do you think could be provided to help you in providing lifestyle advice to cancer patients in the radiotherapy department? Please select all that apply

- [ ] Online training
- [ ] In house training
- [ ] Mandatory CPD training
- [ ] Role expansion
- [ ] Provision of education resources for patients within the department
- [ ] Referral pathways for lifestyle support

Other (please specify)

* 13. How helpful do you think an <u>online</u> training course specifically for therapeutic radiographers on delivering health promotion to cancer patients would be?

- ( ) Extremely Helpful
- ( ) Somewhat Helpful
- ( ) Neither
- ( ) Somewhat Unhelpful
- ( ) Extremely Unhelpful

Health Promotion among Radiographers

* 14. How helpful do you think a face to face training course on delivering health promotion as part of your role to cancer patients would be?

- ◯ Extremely Helpful
- ◯ Somewhat Helpful
- ◯ Neither
- ◯ Somewhat Unhelpful
- ◯ Extremely Unhelpful

Other (please specify)

* 15. If you were provided with a training course what topics would you like delivered? Please select all that apply

- ☐ Video examples of how to deliver advice within my daily role
- ☐ Role play of having a conversation with a patient
- ☐ The current evidence for specific lifestyle behaviours and cancer outcomes
- ☐ Information of available support and patient education resources
- ☐ Other (please specify)

* 16. Would you be interested in a booklet containing relevant lifestyle and behaviour change information that you could give to your patients?

- ◯ Yes
- ◯ No

* 17. From your experience which format do you think patients would like lifestyle information in? Please select all that apply

- ☐ Leaflet/booklet
- ☐ Website
- ☐ mobile app
- ☐ Face-to-Face

Other (please specify)

18. Would you be willing for us to contact you about taking part in a follow-up interview to this questionnaire?

- ◯ Yes
- ◯ No

If you are willing for us to contact you, please provide name and email address and/or contact number

19. We are planning to develop a lifestyle training intervention to support therapeutic radiographers in providing lifestyle advice to cancer patients. Would you be willing for us to contact you in the future about the possibility of being involved in this?

- ◯ Yes
- ◯ No

If you are willing for us to contact you, please provide name, email address and/or contact number

20. Do you have any other comments, questions, or concerns?

Thank you very much for taking the time to complete this Survey.
